# Methyl (4-bromo­benzene­sulfonamido)acetate

**DOI:** 10.1107/S1600536808037550

**Published:** 2008-11-20

**Authors:** Muhammad Nadeem Arshad, M. Nawaz Tahir, Islam Ullah Khan, Ejaz Ahmad, Muhammad Shafiq

**Affiliations:** a Government College University, Materials Chemistry Laboratory, Department of Chemistry, Lahore, Pakistan; b University of Sargodha, Department of Physics, Sargodha, Pakistan

## Abstract

The title compound, C_9_H_10_BrNO_4_S, is an inter­mediate for the formation of benzothia­zines. In the crystal structure, inter­molecular N—H⋯O hydrogen bonds link the mol­ecules, forming *R*_2_^2^(10) ring motifs, which are linked into a two-dimensional polymeric sheet through inter­molecular C—H⋯O hydrogen bonds.

## Related literature

For general background, see: Arshad *et al.* (2008 ▶); Tahir *et al.* (2008 ▶). For a related structure, see: Bornaghi *et al.* (2005 ▶). For ring motifs, see: Bernstein *et al.* (1995 ▶). For bond-length data, see: Allen *et al.* (1987 ▶).
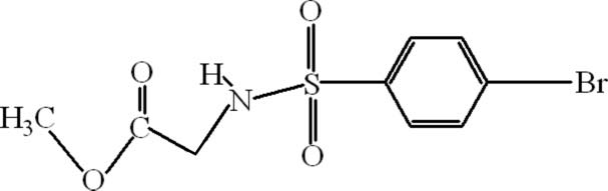


## Experimental

### 

#### Crystal data

C_9_H_10_BrNO_4_S
*M* *_r_* = 308.15
Triclinic, 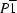

*a* = 6.0451 (2) Å
*b* = 7.0369 (2) Å
*c* = 13.8695 (5) Å
α = 83.866 (2)°
β = 81.190 (1)°
γ = 87.027 (2)°
*V* = 579.31 (3) Å^3^
*Z* = 2
Mo *K*α radiation
μ = 3.73 mm^−1^
*T* = 296 (2) K
0.23 × 0.18 × 0.12 mm

#### Data collection

Bruker Kappa APEXII CCD diffractometer
Absorption correction: multi-scan (*SADABS*; Bruker, 2005 ▶) *T*_min_ = 0.449, *T*_max_ = 0.639
13148 measured reflections
2846 independent reflections
1893 reflections with *I* > 2σ(*I*)
*R*_int_ = 0.038

#### Refinement

*R*[*F* ^2^ > 2σ(*F* ^2^)] = 0.040
*wR*(*F* ^2^) = 0.116
*S* = 1.04
2846 reflections
145 parameters
H-atom parameters constrained
Δρ_max_ = 0.53 e Å^−3^
Δρ_min_ = −0.59 e Å^−3^

### 

Data collection: *APEX2* (Bruker, 2007 ▶); cell refinement: *SAINT* (Bruker, 2007 ▶); data reduction: *SAINT*; program(s) used to solve structure: *SHELXS97* (Sheldrick, 2008 ▶); program(s) used to refine structure: *SHELXL97* (Sheldrick, 2008 ▶); molecular graphics: *ORTEP-3 for Windows* (Farrugia, 1997 ▶) and *PLATON* (Spek, 2003 ▶); software used to prepare material for publication: *WinGX* (Farrugia, 1999 ▶) and *PLATON*.

## Supplementary Material

Crystal structure: contains datablocks global, I. DOI: 10.1107/S1600536808037550/hk2573sup1.cif

Structure factors: contains datablocks I. DOI: 10.1107/S1600536808037550/hk2573Isup2.hkl

Additional supplementary materials:  crystallographic information; 3D view; checkCIF report

## Figures and Tables

**Table 1:** Hydrogen-bond geometry (Å, °)

*D*—H⋯*A*	*D*—H	H⋯*A*	*D*⋯*A*	*D*—H⋯*A*
N1—H1⋯O2^i^	0.86	2.41	2.987 (4)	125
C3—H3*A*⋯O3^ii^	0.97	2.50	3.410 (4)	156

Symmetry codes: (i) 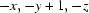; (ii) 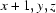.

**Fig. 1.**
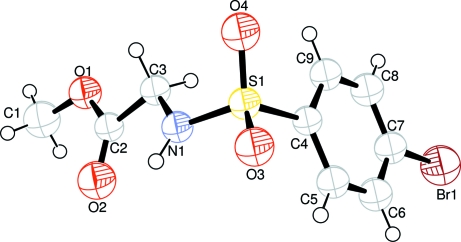
The molecular structure of the title molecule, with the atom-numbering scheme.

**Fig. 2.**
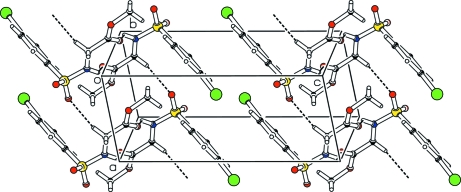
A partial packing diagram. Hydrogen bonds are shown as dashed lines.

## supplementary crystallographic information

### Comment

The glycine methyl ester is the simplest among the amino acids and the *p*-bromobenzenesulfonyl chloride is also comercially available. We have synthesized the title compound, (I), from their condensation. It has been prepared as an intermediate for the formation of benzothiazines, which are of our research interest (Arshad *et al.*, 2008; Tahir *et al.*, 2008).

In the molecule of the title compound, (I), (Fig. 1) the bond lengths (Allen *et al.*, 1987) and angles are within normal ranges, and are comparable with the corresponding values in *N*-(2-nitrophenylsulfonyl)glycine methyl ester, (II), (Bornaghi *et al.*, 2005). Ring A (C4-C9) is, of course, planar, and the Br atom lies slightly out of the ring plane [0.069 (3) Å]. The S1-N1 bond is almost perpendicular to the ring plane, with N1-S1-C4-C5 [-83.4 (3)°] and/or N1/S1/C4/C9 [93.7 (3)°] torsion angles. The (O1/O2/C1-C3) moiety is planar, and it is oriented with respect to ring A at a dihedral angle of 87.74 (3)°.

In the crystal structure, intermolecular N-H···O hydrogen bonds (Table 1) link the molecules to form *R*_2_^2^(10) ring motifs (Bernstein *et al.*, 1995), in which they are linked to form a two dimensional polymeric sheet through intermolecular C-H···O hydrogen bonds (Table 1, Fig. 2).

### Experimental

Glycine methyl ester hydrochloride (0.246 g, 1.95 mmol) was dissolved in water (10 ml) in round bottom flask. The pH of the solution was adjusted to 8–9 using sodium carbonate (1 N), and then 4-bromo benzene sulfonyl chloride (0.5 g, 1.95 mmol) was added. The mixture was stirred for 2 h at room temperature. During the reaction, pH was strictly maintained at 8–9 as HCl produced, which lowers the pH. Colorless solid product obtained was washed, dried and recrystalized from methanol for X-ray analysis (m.p. 393-394 K).

### Refinement

H atoms were positioned geometrically, with N-H = 0.86 Å (for NH) and C-H = 0.93, 0.97 and 0.96 Å for aromatic, methylene and methyl H, respectively, and constrained to ride on their parent atoms with U_iso_(H) = xU_eq_(C,N), where x = 1.5 for methyl H and x = 1.2 for all other H atoms.

### Crystal data

**Table:**

C_9_H_10_BrNO_4_S	*Z* = 2
*M**_r_* = 308.15	*F*_000_ = 308
Triclinic, *P*1	*D*_x_ = 1.767 Mg m^−^^3^
Hall symbol: -P 1	Mo *K*α radiation λ = 0.71073 Å
*a* = 6.0451 (2) Å	Cell parameters from 2846 reflections
*b* = 7.0369 (2) Å	θ = 1.5–28.3º
*c* = 13.8695 (5) Å	µ = 3.73 mm^−^^1^
α = 83.866 (2)º	*T* = 296 (2) K
β = 81.190 (1)º	Prism, colorless
γ = 87.027 (2)º	0.23 × 0.18 × 0.12 mm
*V* = 579.31 (3) Å^3^	

### Data collection

**Table:**

Bruker Kappa APEXII CCD diffractometer	2846 independent reflections
Radiation source: fine-focus sealed tube	1893 reflections with *I* > 2σ(*I*)
Monochromator: graphite	*R*_int_ = 0.038
Detector resolution: 7.40 pixels mm^-1^	θ_max_ = 28.3º
*T* = 296(2) K	θ_min_ = 1.5º
ω scans	*h* = −8→8
Absorption correction: multi-scan(SADABS; Bruker, 2005)	*k* = −9→9
*T*_min_ = 0.449, *T*_max_ = 0.639	*l* = −18→18
13148 measured reflections	

### Refinement

**Table:**

Refinement on *F*^2^	Secondary atom site location: difference Fourier map
Least-squares matrix: full	Hydrogen site location: inferred from neighbouring sites
*R*[*F*^2^ > 2σ(*F*^2^)] = 0.040	H-atom parameters constrained
*wR*(*F*^2^) = 0.116	*w* = 1/[σ^2^(*F*_o_^2^) + (0.0448*P*)^2^ + 0.5395*P*] where *P* = (*F*_o_^2^ + 2*F*_c_^2^)/3
*S* = 1.04	(Δ/σ)_max_ < 0.001
2846 reflections	Δρ_max_ = 0.53 e Å^−^^3^
145 parameters	Δρ_min_ = −0.59 e Å^−^^3^
Primary atom site location: structure-invariant direct methods	Extinction correction: none

### Special details

**Table:**

Geometry. All e.s.d.'s (except the e.s.d. in the dihedral angle between two l.s. planes) are estimated using the full covariance matrix. The cell e.s.d.'s are taken into account individually in the estimation of e.s.d.'s in distances, angles and torsion angles; correlations between e.s.d.'s in cell parameters are only used when they are defined by crystal symmetry. An approximate (isotropic) treatment of cell e.s.d.'s is used for estimating e.s.d.'s involving l.s. planes.
Refinement. Refinement of *F*^2^ against ALL reflections. The weighted *R*-factor *wR* and goodness of fit *S* are based on *F*^2^, conventional *R*-factors *R* are based on *F*, with *F* set to zero for negative *F*^2^. The threshold expression of *F*^2^ > σ(*F*^2^) is used only for calculating *R*-factors(gt) *etc*. and is not relevant to the choice of reflections for refinement. *R*-factors based on *F*^2^ are statistically about twice as large as those based on *F*, and *R*- factors based on ALL data will be even larger.

### Fractional atomic coordinates and isotropic or equivalent isotropic displacement parameters (Å^2^)

**Table:**

	*x*	*y*	*z*	*U*_iso_*/*U*_eq_	
Br1	0.31108 (8)	0.18201 (7)	0.47759 (3)	0.0859 (2)	
S1	−0.15899 (12)	0.80316 (11)	0.19808 (6)	0.0428 (2)	
O1	0.4939 (4)	0.7999 (3)	−0.06466 (17)	0.0535 (6)	
O2	0.2102 (5)	0.6116 (4)	−0.06458 (19)	0.0694 (8)	
O3	−0.3871 (3)	0.7563 (4)	0.20253 (19)	0.0589 (6)	
O4	−0.1032 (4)	0.9893 (3)	0.21559 (19)	0.0602 (6)	
N1	−0.0358 (4)	0.7668 (4)	0.08966 (18)	0.0426 (6)	
H1	−0.1060	0.7168	0.0497	0.051*	
C1	0.6104 (7)	0.7313 (6)	−0.1532 (3)	0.0720 (12)	
H1A	0.7505	0.7935	−0.1714	0.108*	
H1B	0.5207	0.7589	−0.2048	0.108*	
H1C	0.6379	0.5957	−0.1425	0.108*	
C2	0.2962 (5)	0.7298 (4)	−0.0293 (2)	0.0411 (7)	
C3	0.1966 (5)	0.8203 (5)	0.0605 (2)	0.0427 (7)	
H3A	0.2818	0.7791	0.1133	0.051*	
H3B	0.2032	0.9583	0.0477	0.051*	
C4	−0.0381 (5)	0.6363 (4)	0.2804 (2)	0.0385 (6)	
C5	−0.1158 (6)	0.4529 (5)	0.2982 (2)	0.0482 (8)	
H5	−0.2363	0.4206	0.2696	0.058*	
C6	−0.0151 (6)	0.3180 (5)	0.3582 (2)	0.0534 (8)	
H6	−0.0672	0.1944	0.3709	0.064*	
C7	0.1645 (6)	0.3690 (6)	0.3992 (2)	0.0528 (9)	
C8	0.2420 (6)	0.5515 (6)	0.3828 (3)	0.0549 (9)	
H8	0.3625	0.5834	0.4115	0.066*	
C9	0.1398 (5)	0.6868 (5)	0.3233 (2)	0.0484 (8)	
H9	0.1898	0.8111	0.3121	0.058*	

### Atomic displacement parameters (Å^2^)

**Table:**

	*U*^11^	*U*^22^	*U*^33^	*U*^12^	*U*^13^	*U*^23^
Br1	0.0822 (3)	0.1005 (4)	0.0689 (3)	0.0267 (3)	−0.0172 (2)	0.0133 (2)
S1	0.0313 (4)	0.0443 (5)	0.0512 (5)	−0.0016 (3)	−0.0027 (3)	−0.0019 (3)
O1	0.0403 (12)	0.0621 (15)	0.0588 (14)	−0.0191 (10)	0.0057 (10)	−0.0201 (12)
O2	0.0744 (17)	0.0703 (18)	0.0649 (16)	−0.0429 (14)	0.0103 (13)	−0.0240 (14)
O3	0.0288 (11)	0.0740 (17)	0.0704 (16)	−0.0032 (10)	−0.0048 (10)	0.0059 (13)
O4	0.0603 (15)	0.0420 (14)	0.0770 (18)	0.0016 (11)	−0.0034 (13)	−0.0112 (12)
N1	0.0335 (13)	0.0531 (16)	0.0423 (14)	−0.0158 (11)	−0.0078 (10)	0.0004 (12)
C1	0.060 (2)	0.078 (3)	0.076 (3)	−0.016 (2)	0.020 (2)	−0.031 (2)
C2	0.0409 (16)	0.0361 (16)	0.0465 (17)	−0.0116 (12)	−0.0062 (13)	−0.0003 (13)
C3	0.0363 (16)	0.0476 (18)	0.0453 (17)	−0.0140 (13)	−0.0052 (12)	−0.0053 (14)
C4	0.0320 (14)	0.0470 (17)	0.0359 (15)	−0.0035 (12)	−0.0008 (11)	−0.0067 (13)
C5	0.0478 (18)	0.052 (2)	0.0467 (18)	−0.0114 (15)	−0.0110 (14)	−0.0048 (15)
C6	0.061 (2)	0.050 (2)	0.0488 (19)	−0.0070 (16)	−0.0077 (16)	−0.0017 (15)
C7	0.0484 (19)	0.071 (2)	0.0348 (16)	0.0117 (17)	0.0007 (14)	−0.0016 (15)
C8	0.0398 (17)	0.076 (3)	0.050 (2)	−0.0057 (16)	−0.0110 (14)	−0.0062 (18)
C9	0.0386 (17)	0.055 (2)	0.0524 (19)	−0.0110 (14)	−0.0041 (14)	−0.0102 (16)

### Geometric parameters (Å, °)

**Table:**

Br1—C7	1.889 (3)	C3—N1	1.458 (4)
S1—O4	1.424 (2)	C3—H3A	0.9700
S1—O3	1.425 (2)	C3—H3B	0.9700
S1—N1	1.613 (3)	C4—C5	1.381 (4)
S1—C4	1.759 (3)	C4—C9	1.385 (4)
O1—C2	1.320 (3)	C5—C6	1.374 (5)
O1—C1	1.436 (4)	C5—H5	0.9300
O2—C2	1.188 (4)	C6—C7	1.381 (5)
N1—H1	0.8600	C6—H6	0.9300
C1—H1A	0.9600	C7—C8	1.374 (5)
C1—H1B	0.9600	C8—C9	1.377 (5)
C1—H1C	0.9600	C8—H8	0.9300
C2—C3	1.489 (4)	C9—H9	0.9300
			
O4—S1—O3	120.49 (15)	N1—C3—H3B	109.6
O4—S1—N1	106.96 (14)	C2—C3—H3B	109.6
O3—S1—N1	106.53 (14)	H3A—C3—H3B	108.1
O4—S1—C4	107.89 (15)	C5—C4—C9	120.5 (3)
O3—S1—C4	107.66 (14)	C5—C4—S1	119.4 (2)
N1—S1—C4	106.55 (14)	C9—C4—S1	120.1 (2)
C2—O1—C1	117.5 (3)	C6—C5—C4	120.1 (3)
C3—N1—S1	118.9 (2)	C6—C5—H5	120.0
C3—N1—H1	120.6	C4—C5—H5	120.0
S1—N1—H1	120.6	C5—C6—C7	118.9 (3)
O1—C1—H1A	109.5	C5—C6—H6	120.6
O1—C1—H1B	109.5	C7—C6—H6	120.6
H1A—C1—H1B	109.5	C8—C7—C6	121.6 (3)
O1—C1—H1C	109.5	C8—C7—Br1	119.2 (3)
H1A—C1—H1C	109.5	C6—C7—Br1	119.2 (3)
H1B—C1—H1C	109.5	C7—C8—C9	119.4 (3)
O2—C2—O1	124.5 (3)	C7—C8—H8	120.3
O2—C2—C3	125.0 (3)	C9—C8—H8	120.3
O1—C2—C3	110.4 (2)	C8—C9—C4	119.5 (3)
N1—C3—C2	110.1 (2)	C8—C9—H9	120.2
N1—C3—H3A	109.6	C4—C9—H9	120.2
C2—C3—H3A	109.6		
			
C1—O1—C2—O2	1.4 (5)	C4—C5—C6—C7	−0.4 (5)
C1—O1—C2—C3	−178.3 (3)	C5—C6—C7—C8	1.1 (5)
O2—C2—C3—N1	−9.3 (5)	C5—C6—C7—Br1	−177.6 (3)
O1—C2—C3—N1	170.5 (3)	C6—C7—C8—C9	−0.5 (5)
O4—S1—C4—C5	162.0 (2)	Br1—C7—C8—C9	178.1 (2)
O3—S1—C4—C5	30.5 (3)	C7—C8—C9—C4	−0.6 (5)
N1—S1—C4—C5	−83.4 (3)	C5—C4—C9—C8	1.2 (5)
O4—S1—C4—C9	−20.8 (3)	S1—C4—C9—C8	−175.9 (2)
O3—S1—C4—C9	−152.3 (3)	C2—C3—N1—S1	165.0 (2)
N1—S1—C4—C9	93.7 (3)	O4—S1—N1—C3	44.2 (3)
C9—C4—C5—C6	−0.7 (5)	O3—S1—N1—C3	174.3 (2)
S1—C4—C5—C6	176.5 (3)	C4—S1—N1—C3	−71.0 (3)

### Hydrogen-bond geometry (Å, °)

**Table:**

*D*—H···*A*	*D*—H	H···*A*	*D*···*A*	*D*—H···*A*
N1—H1···O2^i^	0.86	2.41	2.987 (4)	125
C3—H3A···O3^ii^	0.97	2.50	3.410 (4)	156

Symmetry codes: (i) −*x*, −*y*+1, −*z*; (ii) *x*+1, *y*, *z*.
